# Rescuing neuronal cell death by RAIDD- and PIDD- derived peptides and its implications for therapeutic intervention in neurodegenerative diseases

**DOI:** 10.1038/srep31198

**Published:** 2016-08-09

**Authors:** Tae-Ho Jang, In-Hye Lim, Chang Min Kim, Jae Young Choi, Eun-Ae Kim, Tae-Jin Lee, Hyun Ho Park

**Affiliations:** 1 School of Biotechnology and Graduate School of Biochemistry at Yeungnam University, Gyeongsan 38541, South Korea; 2 Department of Anatomy, College of Medicine, Yeungnam University, 317-1 Daemyung-Dong Nam-Gu, Daegu 42415, South Korea

## Abstract

Caspase-2 is known to be involved in oxidative-stress mediated neuronal cell death. In this study, we demonstrated that rotenone-induced neuronal cell death is mediated by caspase-2 activation via PIDDosome formation. Our newly designed TAT-fused peptides, which contains wild-type helix number3 (H3) from RAIDD and PIDD, blocked the PIDDosome formation *in vitro*. Furthermore, peptides inhibited rotenone-induced caspase-2-dependent apoptosis in neuronal cells. These results suggest that PIDD- or RAIDD-targeted peptides might be effective at protecting against rotenone-induced neurotoxicity. Our peptides are novel neuronal cell apoptosis inhibitors that might serve as a prototype for development of drugs for the treatment of neurodegenerative diseases.

Neurodegenerative diseases such as Alzheimer’s, Parkinson’s, and Huntington’s disease are caused by excessive death of the neurons, which mainly occurs via a programmed cell death mechanism known as apoptosis^1^,^2^,^3^. Apoptosis is mediated by the sequential activation of caspases, which are cysteine proteases that use cysteine as a nucleophile. Caspases are classified into two distinct groups according to their sequence of activation, initiator caspases (including caspases 8, 9 and 10) and effector caspases (including caspases 3 and 7)^4^,^5^,^6^,^7^. Initiator caspases contain N-terminal pro-domains for the formation of caspase activating complexes, which can provide proximity to the initiator caspase for self-activation. Effector caspases are activated by activated initiator caspases^8^,^9^,^10^.

Caspase-2, which is the most evolutionarily conserved caspase across animal species, is an enigmatic caspase that has features of both initiator and effector caspases^11^,^12^. Specifically, caspase-2 can be classified as an effector caspase because it shares substrate specificities with effector caspase^13^; however, it can also be classified as an initiator caspase since it contains the N-terminal pro-domain, which mediates protein interaction during the formation of caspase activating complexes and is cleaved by activation^14^. Although the role of caspase-2 is not clear, several recent studies have reported that it acts upstream of the mitochondrial pathway in response to genotoxic stress^15^,^16^. The caspase-2 activating complex known as PIDDosome is composed of three proteins, PIDD, RAIDD, and caspase-2^17^,^18^. PIDDosome independent activation of caspase-2 has been also reported^19^. PIDD contains seven leucine rich repeats (LRRs), two ZU-5 domains and a death domain (DD) at the C-terminus^20^. In addition to its apoptotic role with RAIDD and caspase-2, PIDD also plays a cell survival role via interaction with RIP1, a kinase that has been implicated in the activation of NF-κB^21^,^22^. RAIDD is an adapter protein that contains both caspase recruiting domain (CARD) and death domain (DD) at the N-terminus and C-terminus, respectively^23^. Caspase-2 contains the CARD domain at its N-terminus for protein interaction. The assembly of PIDDosome is mediated by a DD:DD interaction between PIDD and RAIDD, as well as a CARD:CARD interaction between RAIDD and caspase-2. The crystal structure of the PIDDosome core, which is composed of seven RAIDD DD and five PIDD DD molecules, revealed that PIDDosome might be a highly oligomeric complex^24^.

Rotenone has been shown to cause oxidative damage and endoplasmic reticulum stress, as well as lead to apoptosis^25^,^26^. Additionally, chronic exposure of rats to rotenone induces Parkinsonism *in vivo*^27^. Interestingly, it is known that oxidative stress induced by rotenone can activate caspase-2, as well as other pro-apoptotic molecules such as Bax and caspase-3^28^. In addition, loss of caspase-2 markedly inhibited rotenone-induced apoptotic events such as activation of Bid and Bax and the release of cytochrome c and apoptosis inducing factor (AIF) from the mitochondria of neuronal cells^29^. These studies suggested that caspase-2 acts as an initiator caspase to mediate rotenone-induced apoptosis in neuronal cells. In addition, PIDDosome is proposed to be a hypothetical molecular target for therapy against neuronal cell death after transient global cerebral ischemia^30^.

Our previous study showed that synthetic TAT-fused peptides designed by the structure of the RAIDD:PIDD complex play protective roles against genotoxic stress-induced apoptosis in human renal cancer models^31^. However, it is unknown whether PIDD- or RAIDD-targeted peptides can protect against rotenone toxicity in Parkinsonism models. In this study, we designed new TAT-fused peptides that contain wild-type helix number 3 (H3) from RAIDD and PIDD. We demonstrated that newly designed PIDD- or RAIDD-targeted peptides potently inhibited interaction of the PIDD and RAIDD in a dose-dependent manner, with a stronger effect than previously generated peptides that contain dominant-negative mutants. Furthermore, both peptides slightly inhibited rotenone-induced apoptosis and rotenone-mediated caspase-2 activation in SH-SY5Y cells. These findings suggest that PIDD- or RAIDD-targeted peptides might effectively protect against rotenone-induced neurotoxicity. The results presented herein suggest that both peptides are novel neuronal cell apoptosis inhibitors that may serve as prototypes for the therapeutic intervention.

## Results and Discussion

### Rational design of TAT-fused peptides based on the structure of the RAIDD DD:PIDD DD complex

The crystal structure of the RAIDD DD:PIDD DD complex, which is in the core of the PIDDosome, has been elucidated and provided information regarding the interfaces formed by the complex between RAIDD DD and PIDD DD^24^. This information can be used to design small chemicals or peptides that can specifically block formation of the protein complex. We previously generated TAT-fused peptides from RAIDD DD and PIDD DD and tested their effects on formation of the PIDDosome^31^. The results showed that previously generated peptides that include mutations R147E on RAIDD and Y814A on PIDD DD can block PIDDosome formation and rescued genotoxic-stress induced apoptosis in cancer cells. The working concentration of the peptides was around 1 mM.

**Figure 1:**
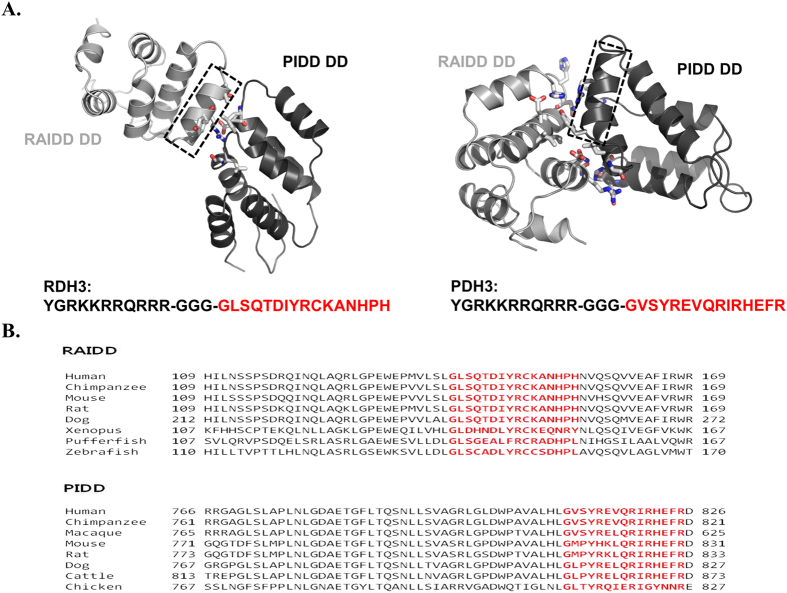
Rational design of TAT-fused peptides based on the structure of the RAIDD DD:PIDD DD complex. (**A**) The location and amino acid sequence of RDH3 and PDH3. Helix3 positions on RAIDD DD (RDH3) and PIDD DD (PDH3) are shown in the black dot-box. The full-sequence of TAT fused peptides, RDH3 and PDH3, are indicated below the cartoon. (**B**) Sequence alignment of RAIDD DD and PIDD DD from different species. The location of Helix 3 (H3) in the sequence of the protein is indicated by red.

To improve the peptide efficiency, we generated TAT-fused peptides including the TAT sequence and wildtype peptide derived from helix3 (H3) of RAIDD DD and PIDD DD (Fig. 1A). Previously conducted structure-based mutagenesis studies have shown that the H3 of both RAIDD DD and PIDD DD is important for complex formation^32^. The TAT sequence is attached to give peptides the potential for cell penetration^33^,^34^. Cell-permeable peptides containing wildtype RAIDD DD H3 (RDH3) and wildtype PIDD H3 (PDH3) fused to the internalization sequence of the TAT transactivator domain (YGRKKRRQRRR) at their N-terminus were synthesized using GGG as a linker on the peptides. The amino acid sequences of H3 were conserved among RAIDD DDs and PIDD DDs across species (Fig. 1B).

### RDH3 and PDH3 block formation of the PIDDosome core *in vitro*

**Figure 2:**
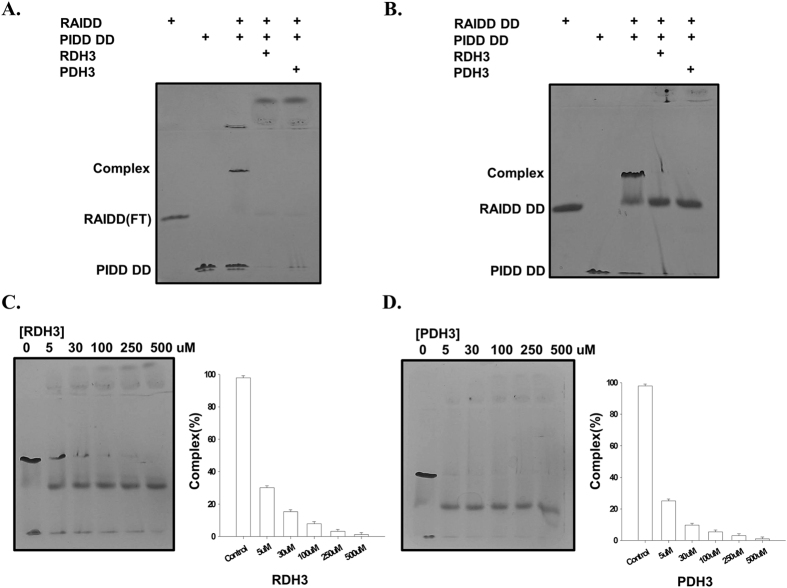
RDH3 and PDH3 block formation of the PIDDosome core *in vitro*. (**A**) Purified RAIDD, PIDD DD, RAIDD + PIDD DD mixture, and RAIDD + PIDD DD mixture + RDH3 or PDH3 mixture were pre-incubated for 1 hour at room temperature, after which the mixtures were subjected to native-PAGE. The complex formation and inhibition by peptides were then evaluated based on the appearance and disappearance of a new complex band on native-PAGE. (**B**) The same native-PAGE experiment was conducted using RAIDD DD instead of full-length RAIDD. (**C**) Peptide dose-dependent inhibition of PIDDosome core formation. The RAIDD DD:PIDD DD complex was prepared and mixed with the concentrations of RDH3 peptide indicated above the gel, after which the mixtures were subjected to native-PAGE. The concentration of peptide stock solution was 500 μM. Stock was diluted as indicated and used for blocking studies. The percentage of the complex was evaluated based on the appearance and thickness of the band generated. The thickness of the complex band formed in the control without any peptide was considered to be 100%. (**D**) The same experiment investigating peptide dose-dependent effect was conducted using PDH3 peptide instead of RDH3 at (**C**).

To investigate the ability of the synthesized peptides to inhibit formation of the PIDDosome *in vitro*, we performed native-PAGE with purified target proteins. Native-PAGE is commonly used to detect protein complexes by analyzing the newly generated bands after mixing two or more proteins. We initially purified full-length RAIDD, RAIDD DD, and PIDD DD using a quick two step chromatography method. Purified PIDD DD was then incubated with either RAIDD or RAIDD DD to produce stable complexes. Next, either RDH3 or PDH3 peptide was added to determine if they could inhibit the complex formation. Full-length RAIDD produced a stable complex band by mixing with PIDD DD on native-PAGE (Fig. 2A). However, the RAIDD:PIDD DD complex bands disappeared after incubation with the TAT-fused peptides, RDH3 and PDH3, demonstrating that both TAT-fused peptides including wildtype RAIDD DD H3 and PIDD DD H3 block the complex formation *in vitro* (Fig. 2A). Formation of the stable complex produced by RAIDD DD and PIDD DD was also inhibited by RDH3 and PDH3 (Fig. 2B). The complex band more clearly disappeared following mixing with either PDH3 or RDH3 (Fig. 2B). Although complex bands were clearly disappeared by adding peptides on native-PAGE, dissociated RAIDD and PIDD were not proportionally detected on the gel (Fig. 2A,B). This may be because dissociated RAIDD and PIDD can form alternative oligomeric complex. Detection of alternative complex band on the top of native-PAGE and decreased full-length RAIDD and PIDD DD bands were supported our speculation. We next investigated the effects of peptides dose. Specifically, we analyzed the decrease in the complex band on native-PAGE in the presence of different concentrations of RDH3 and PDH3. To accomplish this, different concentrations of either RDH3 or PDH3 were added when we produced the RAIDD DD:PIDD DD complex. The mixtures were then loaded onto a native-PAGE gel. As shown in Fig. 2C,D, treatment of a mixture of RAIDD DD and PIDD DD with 30 μM to 500 μM of RDH3 and PDH3 inhibited the complex formation almost completely. To show the clear dose-dependent effect of PDH3, we also performed dose-dependent assay at the low peptide concentration range, from 0.5 uM to 3 uM (Supplementary Figure 1). These experimental data clearly indicated that both RDH3 and PDH3 interfere with the complex formation of the PIDDosome core and this inhibition is dose-dependent.

The peptides specificity was also analyzed by native-PAGE with another DD family, RIP1 DD and FADD DD, which can form RIPoptosome core by direct interaction^35^. Native-PAGE analysis showed that not only individual RIP DD DD and FADD DD but also complex of RIP1 DD:FADD DD were not effect by adding both peptides, RDH3 and PDH3 (Supplementary Figure 2), indicating that RDH3 and PDH3 can specifically disrupt the complex formation of RAIDD DD and PIDD DD.

**Figure 3:**
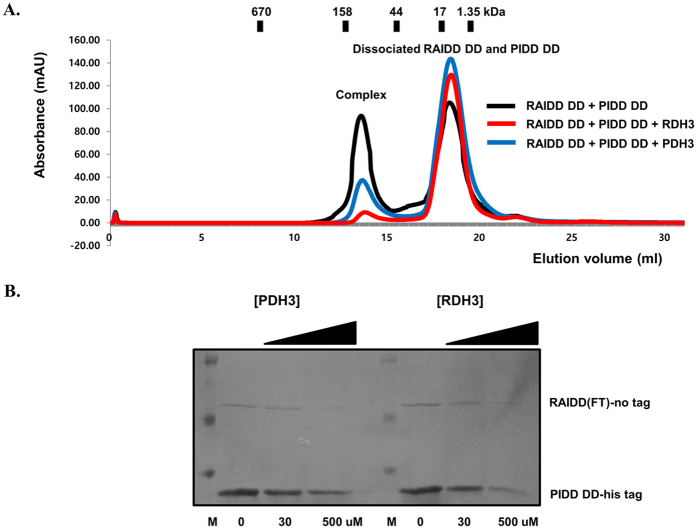
Dose-dependent peptides effect was confirmed with size-exclusion chromatography and pull-down assay. (**A**) RAIDD DD + PIDD DD mixture and RAIDD DD + PIDD DD mixture + RDH3 or PDH3 were pre-incubated for 1 hour at room temperature, after which the mixtures were subjected to size-exclusion chromatography. The complex formation and inhibition effect by peptides were then evaluated based on the appearance and height of peak on the profile. (**B**) Full-down assay with peptides. Full-length RAIDD without any tag was mixed and incubated with his-tagged PIDD DD, Ni-NTA beads and either RDH3 or PDH3. Amount of peptides added for the experiment is indicated.

To confirm the peptide effect on the complex formation, we also performed size-exclusion chromatography with RAIDD DD and PIDD DD mixture in the presence of either 100 μM RDH3 or PDH3. As shown in Fig. 3A, the complex peak was dramatically reduced by adding peptides, while dissociated RAIDD DD and PIDD DD peak was increased. *In vitro* full-down assay also showed that the complex formation can be blocked by peptides (Fig. 3B). Amount of pull-downed RAIDD was reduced by incubating with either 30 μM RDH3 or PDH3 and completely disappeared by incubating with either 500 μM RDH3 or PDH3. Based on all the *in vitro* analysis, we concluded that RDH3 and PDH3 can inhibit formation of PIDDosome core complex.

### Inhibition capacity of wild type peptides is stronger than that of mutant peptides

**Figure 4:**
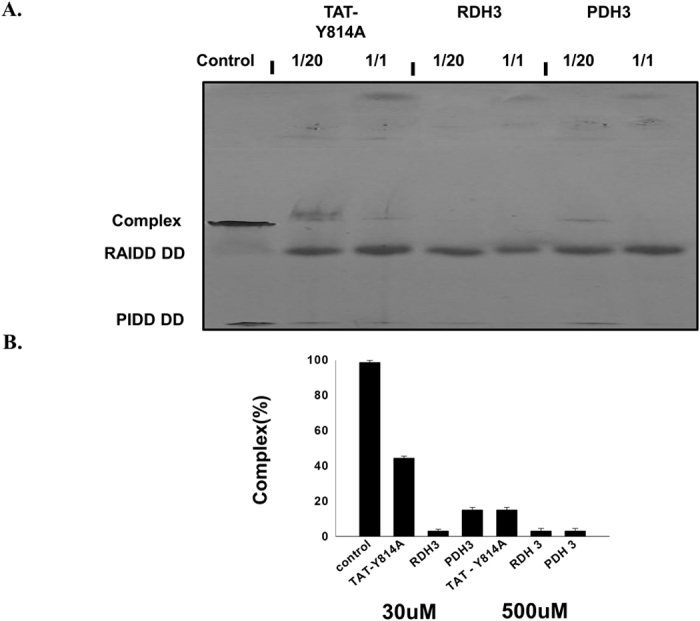
Inhibition capacity of wild type peptides is stronger than that of mutant peptides. Purified RAIDD DD and PIDD DD were mixed and incubated with the indicated peptides, after which the mixtures were subjected to native-PAGE. Complex formation and inhibition by peptides were then evaluated based on the appearance and disappearance of a new complex band on native-PAGE. The concentration of peptide stock solution was 500 μM. Stock was diluted as indicated and used for complex blocking studies. (**B**) Bar graph presentation for better visualization of the results of (**A**). The percentage of the complex was evaluated based on the appearance and thickness of the band generated. The thickness of the complex band formed in the control without any peptide was considered to be 100%. Values are means SD of n = 3.

We previously reported that TAT-fused peptides generated by RAIDD DD and PIDD DD that included mutations at R147E for RAIDD peptide (TAT-R147E) and Y814A for PIDD peptide (TAT-Y814A) exhibited inhibitory effects against genotoxic stress-triggered apoptosis of cancer cells by blocking PIDDosome formation^31^. Because we observed *in vitro* potency of the newly generated peptides, RDH3 and PDH3, we investigated the capacity of the newly generated peptides relative to the previously generated peptide TAT-Y814A. To compare the inhibitory effects between previous TAT-Y814A peptide and current wildtype peptides against formation of the PIDDosome core, we performed a native-PAGE experiment. As expected, we found that the inhibitory capacity of RDH3 and PDH3 was more than five times stronger than that of TAT-Y814A (Fig. 4A,B). While 30 μM of TAT-Y814A peptide blocked around 50% of the complex formation, RDH3 and PDH3 completely blocked complex formation at 30 μM. These findings indicate that current peptides, which are designed by the wild type H3 peptide of RAIDD and PIDD (RDH3 and PDH3, respectively) exert more powerful effects than previously generated peptides. Because the dose should be considered prior to application for medical purposes, RDH3 and PDH3 peptides have the potential for use in development of anti-apoptotic drugs.

### Rotenone induced cell death in a dose-dependent manner in human neuroblastoma cells

**Figure 5:**
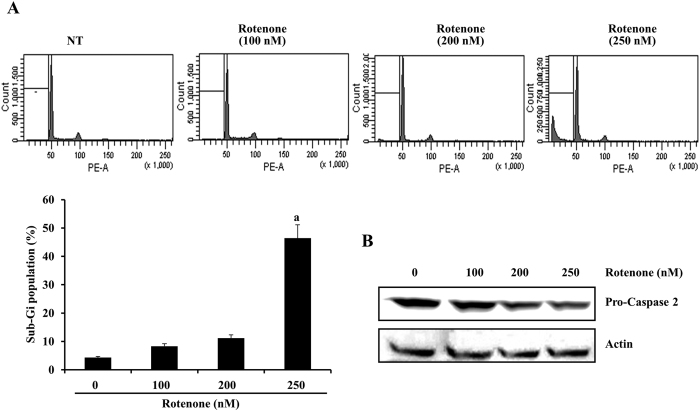
Rotenone treatment induces cell death in a dose-dependent manner in SH-SY5Y cells. (**A**,**B**) Flow cytometric analysis of apoptotic cells (histogram). SH-SY5Y cells were treated with the indicated concentrations of rotenone for 48 h. Apoptosis was analyzed as a sub-G1 fraction by FACS. Representative data from one of three experiments yielding similar results are shown. Flow cytometric analysis of apoptotic cells (Graph). ^a^p < 0.05 compared to non-treated (NT) cells. (**C**) Rotenone treatment induced cleavage of procaspase-2 protein. SH-SY5Y cells were incubated with the indicated concentrations of rotenone for 48 h. Equal amounts of cell lysates (40 μg) were subjected to electrophoresis and analyzed by Western blot for procaspase-2 antibodies. Western blotting of actin levels was included to show that equivalent amounts of protein were loaded in each lane.

To investigate the anti-apoptotic effects of RDH3 and PDH3 peptides in disease models, we evaluated the rotenone-induced cellular toxicities and activation of the procaspase-2 system in SH-SY5Y cells. To accomplish this, we first determined cell death using flow cytometry analysis to detect the hypodiploid cell populations. As shown in Fig. 5A, treatment of SH-SY5Y cells with rotenone resulted in markedly increased accumulation of sub-G1 phase cells in a dose-dependent manner. After 48 h of exposure to rotenone, the levels of procaspase-2 decreased progressively with increasing concentrations of rotenone (Fig. 5B), suggesting that rotenone induced procaspase-2 activation in SH-SY5Y cells. Since caspase-2 dependent cell death was detected in several neuronal cells^29^, rotenone-induced cell death by activation of caspase-2 was not surprised.

### RDH3 and PDH3 partially inhibit apoptosis induced by rotenone treatment in SH-SY5Y cells

**Figure 6:**
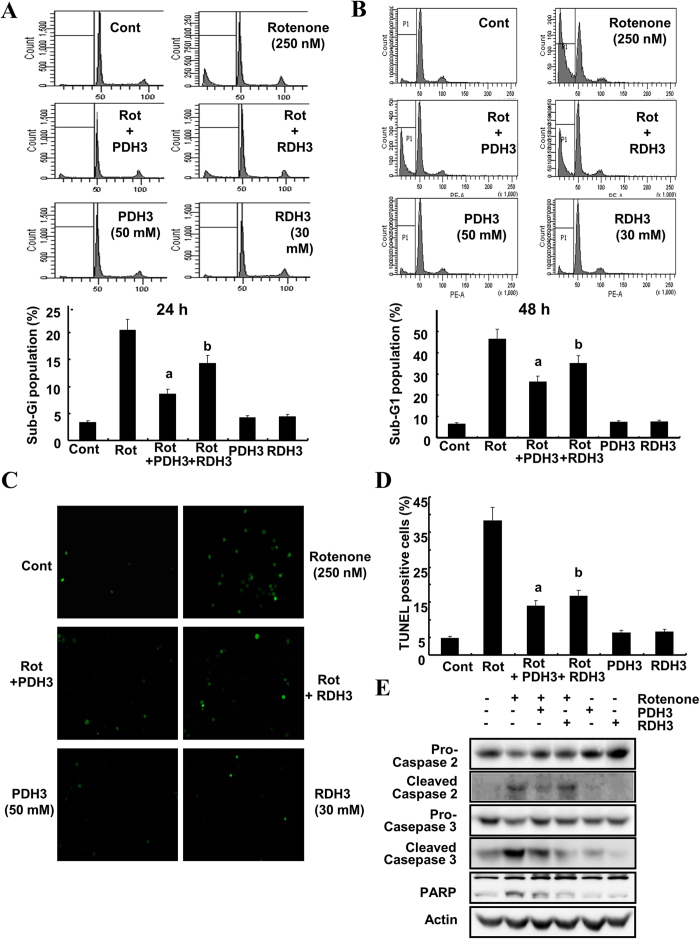
RDH3 and PDH3 partly inhibit apoptosis induced by rotenone treatment of SH-SY5Y cells. (**A**,**B**) RDH3 and PDH3 partly inhibit apoptosis induced by rotenone treatment in SH-SY5Y cells. After TAT fused peptides were pre-incubated for 1 hour, the cells were treated with rotenone. After 24 h (**A**) or 48 h (**B**), apoptosis was analyzed as a sub-G1 fraction by FACS (Top; FACS histogram, Bottom; graph of sub-G1 population). Data shown are the means ± SD (n = 3). a, bp < 0.05 for rotenone-treated cells versus rotenone + PDH3-, or rotenone + RDH3-treated cells by ANOVA. (**C**,**D**) Rotenone-induced DNA fragmentation was reduced by RDH3 and PDH3. DNA fragmentation was measured by TUNEL staining of SH-SY5Y cells. Representative pictures show TUNEL staining (**C**). A DNA fragmentation detection kit was used to measure the fragmented DNA (**D**). (**E**) RDH3 and PDH3 partly suppress cleavage of procaspase-2 induced by rotenone treatment in SH-SY5Y cells. After TAT fused peptides were pre-incubated for 1 hour, the cells were treated with rotenone for 48 h. Equal amounts of cell lysates (40 μg) were subjected to electrophoresis and analyzed by Western blot for caspase-2, caspase-3, and PARP. Actin was used for normalization.

We demonstrated the ability of transduced recombinant RDH3 and PDH3 to inhibit the intrinsic apoptotic pathway induced by 250 nM rotenone and assessed the level of apoptotic cells by flow cytometry in SH-SY5Y cells. One hour after pre-incubation with 50 mM RDH3 and 30 mM PDH3, cells were stimulated concomitantly with rotenone for 24 h or 48 h. As shown in Fig. 6A–D, RDH3 and PDH3 slightly reduced rotenone-induced cell death and DNA fragmentation, which are hallmarks of apoptotic cells. Treatment of SH-SY5Y cells with rotenone resulted in a marked increase in the cleavage of procaspase-2 (Fig. 6E), which was partly attenuated by RDH3 and PDH3. These results indicate that interaction between PIDD and RAIDD was involved in rotenone-induced cell death and activation of procaspase-2 in SH-SY5Y cells. Since caspases are the major executioners of apoptosis^4^,^5^,^6^,^7^, we determined whether caspase-2 acts as an initiator caspase or effector caspase in rotenone-treated neural cells. As shown in Fig. 6E, transduced recombinant RDH3 and PDH3 suppressed cleavage of procaspase-3 and PARP, known to substrate for caspase-3. This result suggests the possibility that inhibition of interaction between PIDD and RAIDD attenuate procaspase-3 activation as well as that caspase-2 act as upstream caspase to mediate rotenone-induced apoptosis in our system. To test the capability and ability of the TAT fused peptides to cross the cell membranes, the cells were inspected via fluorescence microscopy for the specific intracellular accumulation of a fluorescent signal after 24 h of incubation. RDH3 and PDH3 efficiently transduced SH-SY5Y cell lines to a level of almost 100% after 24 h of incubation (data not shown).

### Suppression of PIDD or RAIDD expression attenuate rotenone-induced cell death in SH-SY5Y cells

**Figure 7:**
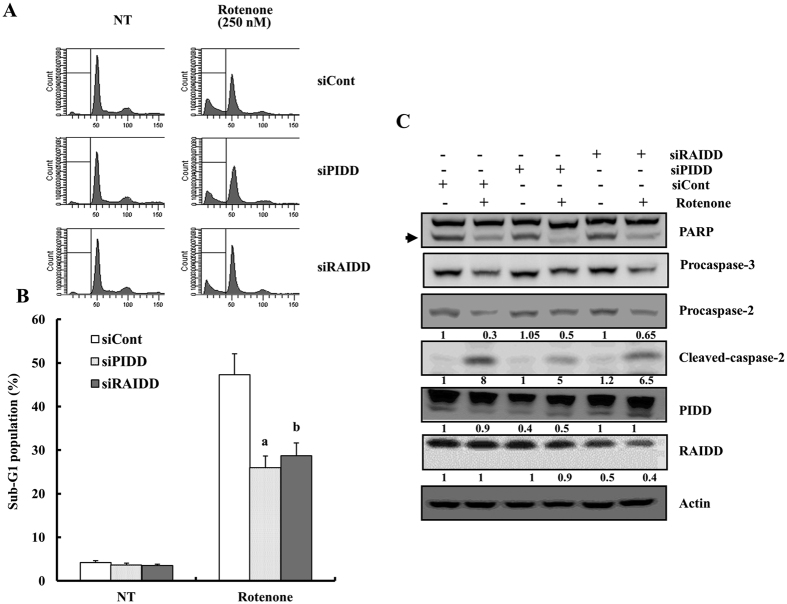
Suppression of PIDD or RAIDD expression attenuates rotenone-induced cell death in SH-SY5Y cells. (**A**,**B**) After SH-SY5Y cells were transfected with PIDD or RAIDD siRNAs or non-targeting siRNA for 24 h, cells were treated with rotenone for 48 h. Flow cytometric analysis of apoptotic cells (histogram) (**A**) and bar graph of sub-G1 population (**B**) are shown. NT indicates non-treated cells. Data shown are the means ± SD (n = 3). ^a,b^p < 0.05 for siCont-transfeced cells versus siPDH3-, or siRDH3-transfected cells by ANOVA. (**C**) Equal amounts of cell lysates (40 μg) were subjected to electrophoresis and analyzed by Western blot for PARP and caspase-2 antibodies. Western blotting of actin levels was included to show that equivalent amounts of protein were loaded in each lane. Relative levels of each protein in drug-treated cells were expressed as a ratio of the densitometric value of each protein to that of Actin using the Bio-Rad Gel Doc System. Arrow indicates cleaved PARP.

SH-SY5Y cells were transiently transfected with PIDD or RAIDD siRNAs to determine whether PIDD or RAIDD are responsible for the rotenone-induced cell death. As shown in Fig. 7A,B, silencing PIDD or RAIDD attenuated rotenone-induced cell death when compared to cells transfected with control siRNA. Next, we checked whether knockdown of PIDD or RAIDD could attenuate rotenone-mediated cleavage of procaspase-2. As shown in Fig. 7C, suppression of PIDD or RAIDD expression by siRNA reduced rotenone-induced procaspase-2 cleavage in SH-SY5Y cells. Western blotting analysis demonstrated that PIDD or RAIDD siRNA potently down-regulated the expression of PIDD and RAIDD (Fig. 7C). Taken together, these findings suggest that PIDD or RAIDD play an important role in rotenone-induced cell death in SH-SY5Y cells, and that RDH3 and PDH3 can block the complex formation between PIDD and RAIDD. Additionally, we found that knockdowns of PIDD or RAIDD expression siRNAs reduced rotenone-induced procaspase-3 and PARP cleavages, suggesting that caspase-2 works as an upstream activator of caspase-3 in neuronal cells (Fig. 7C). Further studies are needed to prove the PIDDosome formation after rotenone treatment in our system.

### Suppression of PIDD or RAIDD expression attenuate rotenone- or 3-nitropropionic acid-induced cell death in PC12 cells

**Figure 8:**
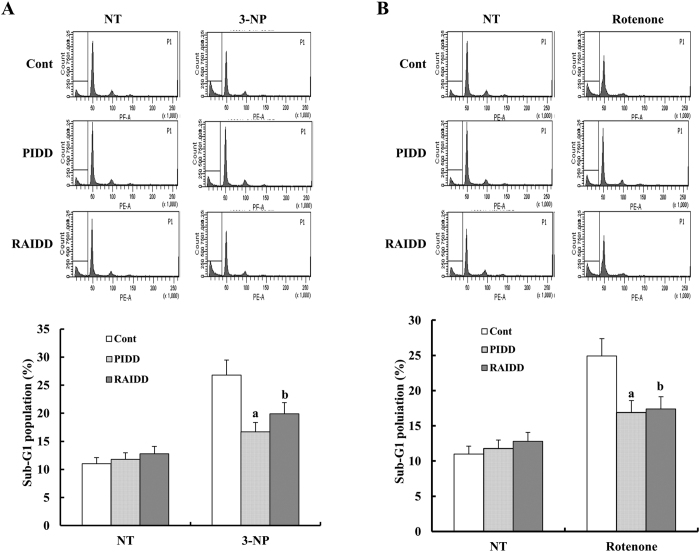
RDH3 and PDH3 partly inhibit apoptosis induced by 3-NP or rotenone treatment of PC12 cells. (**A**) RDH3 and PDH3 partly inhibit apoptosis induced by 3-NP treatment in PC12 cells. After TAT fused peptides were pre-incubated for 1 hour, the cells were treated with 3-NP. After 24 h, apoptosis was analyzed as a sub-G1 fraction by FACS (Top; FACS histogram, Bottom; graph of sub-G1 population). Data shown are the means ± SD (n = 3). ^a,b^p < 0.05 for 3-NP -treated cells versus 3-NP + PDH3-, or 3-NP + RDH3-treated cells by ANOVA. (**B**) RDH3 and PDH3 partly inhibit apoptosis induced by rotenone treatment in PC12 cells. After TAT fused peptides were pre-incubated for 1 hour, the cells were treated with rotenone. After 24 h, apoptosis was analyzed as a sub-G1 fraction by FACS (Top; FACS histogram, Bottom; graph of sub-G1 population). Data shown are the means ± SD (n = 3). ^a,b^p < 0.05 for rotenone-treated cells versus rotenone + PDH3-, or rotenone + RDH3-treated cells by ANOVA.

Systemic administration of 3-nitropropionic acid (3-NP), a specific inhibitor of mitochondrial respiratory complex II, can cause Huntington’s Disease (HD)-like neuropathology in animals^36^,^37^. To check the cytoprotective effects of RDH3 and PDH3 peptides on this neurodegenerative disease model *in vitro*, PC12 cells were stimulated concomitantly with 3-NP or rotenone for 24 h after pre-incubation with 50 μM RDH3 and 30 μM PDH3. As shown in Fig. 8A,B, RDH3 and PDH3 slightly reduced 3-NP- or rotenone-induced cell death in PC12 cells. These results indicate that interaction between PIDD and RAIDD was involved in 3-NP- or rotenone-induced cell death in these cells. Once again, our results indicate that RDH3 and PDH3 might have potential applications as therapeutic agents for treating neurodegenerative diseases such as Huntington’s disease.

## Conclusion

Since the caspase-2 activating complex known as PIDDosome governs genotoxic-stress mediated apoptosis, specific inhibitors that block the formation of PIDDosome have therapeutic potential. Caspase-2 dependent cell death was detected in several neuronal cells, which can cause neurodegenerative diseases^29^. These findings indicate that blocking PIDDosome formation followed by caspase-2 inhibition might be a proper strategy to development of drugs for treatment of neurodegenerative diseases caused by excessive neuronal cell death under certain conditions. To develop chemicals or peptide inhibitors that specifically suppress apoptosis, we have been biochemically and structurally investigating the PIDDosome. Here, we developed novel TAT-fused peptides, RDH3 and PDH3, which exert strong negative effects on PIDDosome formation and can rescue rotenone-treated neuronal cell death. Although it is not clear that the peptides are actually blocking a complex from forming or rather forming an alternative complex in some point, either way partially rescues cell death based on survival cell data. In conclusion, our TAT-fused peptides derived from wildtype RAIDD and PIDD inhibit PIDDosome formation *in vitro* and rescued rotenone-mediated neuronal cell death. These results suggest that RDH3 and PDH3 are highly effective inhibitors of neuronal cell death that might serve as candidates for anti-apoptotic drug development, particularly for treatment of neurodegenerative diseases such as Alzheimer’s, Parkinson’s and Huntington’s disease.

## Methods

### Peptide synthesis

Cell-permeable peptides containing RAIDD helix3 (GLSQTDIY**R**CKANHPHNV) and PIDD helix3 (GVS**Y**REVQRIRHEFR) fused to the internalization sequence of TAT transactivator domain (YGRKKRRQRRR) at their N-termini were synthesized and purified by Peptron (Dae-jeon, South Korea). Linker (GGG) was introduced between the TAT sequence and helix. N-terminally FITC (fluorescein isothiocyanate) attached peptides were synthesized and purified by Peptron (Dae-jeon, South Korea).

### Protein expression and purification

The purification methods have been described in detail elsewhere^24^. RAIDD DD contains amino acids 94–199, PIDD DD contains amino acids 777–883, and full-length RAIDD contains amino acids 1–199. Briefly, recombinant RAIDD DD and PIDD DD were expressed in *E. coli* BL21 (DE3) RILP and purified as previously described^24^. RAIDD was expressed in the BL21 (DE3) *E. coli* line. The purification process for RAIDD was similar to that used for RAIDD DD. Expression was induced by incubation in the presence of 0.5 mM isopropyl-β-D-thiogalactopyranoside (IPTG) overnight at 20 °C. The bacteria were then collected, resuspended and lysed by sonication in 50 ml lysis buffer (20 mM Tris-HCl at pH 7.9, 500 mM NaCl, 20 mM imidazole, and 5 mM β-ME), after which the cell debris was removed by centrifugation at 16,000 rpm for 1 hour at 4 °C. Next, the His-tagged target was purified by affinity chromatography using Ni-NTA beads (Qiagen) followed by gel-filtration chromatography using S-200 (GE healthcare) pre-equilibrated with buffer containing 20 mM Tris-HCl pH 8.0 and 150 mM NaCl.

### Complex dissociation assay by native-PAGE

Formation of the complex was assayed by native (non-denaturing) PAGE conducted on a PhastSystem (GE Healthcare) with pre-made 8–25% acrylamide gradient gels (GE Healthcare). Coomassie brilliant blue was used for staining and detection of the bands. RAIDD DD:PIDD DD and RAIDD:PIDD DD complexes were prepared in Tris buffer (20 mM Tris pH 8.0 and 50 mM NaCl). This complex was then pre-incubated with RDH3 or PDH3 for 1 hour, after which the mixture was subjected to the gel. The percentage of the complex was evaluated based on the appearance of newly formed bands. The amount of the complex formed without peptides was considered to be 100%.

### Complex dissociation assay by size-exclusion chromatography

Similar amount of purified RAIDD DD and PIDD DD mixed and incubated with100 μM of either RDH3 or PDH3 for 1 hour on room temperature. The mixture was then loaded onto S-200 column (GE healthcare) pre-equilibrated with buffer containing 20 mM Tris-HCl pH 8.0 and 150 mM NaCl.

### Pull-down assay

Full-length RAIDD without any tag was mixed and incubated with his-tagged PIDD DD, Ni-NTA beads and either RDH3 or PDH3. Incubated mixture was applied to a gravity-flow column (Bio-Rad). The beads was washed using 5 ml washing buffer (20 mM Tris-HCl pH 7.9, 500 mM NaCl, 30 mM imidazole). The Ni-NTA bound protein was eluted from the column using an elution buffer (20 mM Tris-HCl pH 7.9, 500 mM NaCl, 250 mM imidazole) and analyzed by SDS-PAGE.

### Cell culture and immunoblotting

SH-SY5Y human neuroblastoma cells were obtained from the Korean Cell Line Bank (KCLB, Seoul, Korea). Cells were cultured in DMEM medium supplemented with 10% fetal calf serum and penicillin-streptomycin. PC12 cells (Korean Cell Line Bank, Korea) were cultured in RPMI 1640 with 10% fetal bovine serum (FBS) at 37 °C with 5% CO_2_. For the experiments, cells were seeded at a density of 2 × 10^6^ cells per well. Investigation of the rotenone-induced apoptosis was conducted by pre-incubating cells with TAT fusion proteins (50 μM or 30 μM) such as PDH3 and RDH3 for 30 min prior to the addition of apoptotic stimulus for different times. Cellular lysates were prepared by suspending 2 × 10^6^ cells in 100 μl of lysis buffer (137 mM NaCl, 15 mM EGTA, 0.1 mM sodium orthovanadate, 15 mM MgCl_2_, 0.1% Triton X-100, 25 mM MOPS, 100 μM phenylmethylsulfonyl fluoride, and 20 μM leupeptin, adjusted to pH 7.9). The cells were then disrupted by sonication and extracted at 4 °C for 30 min, after which the proteins were electro-transferred to Immobilon-P membranes (Millipore Corporation, Bedford, MA, USA). Detection of specific proteins was conducted using an ECL Western blotting kit according to the manufacturer’s instructions.

### Flow Cytometry Analysis

Apoptosis was quantified by fluorescence-activated cell sorting (FACS) analysis. Briefly, approximately 1 × 10^6^ SH-SY5Y cells were suspended in 100 μl of PBS, after which 200 μl of 95% ethanol were added and the samples were vortexed. The cells were then incubated at 4 °C for 1 h, washed with PBS, and resuspended in 250 μl of 1.12% sodium citrate buffer (pH 8.4) with 12.5 μg of RNase. Incubation was continued at 37 °C for 30 min. The cellular DNA was then stained by applying 250 μl of propidium iodide (50 μg/ml) for 30 min at room temperature. Finally, the stained cells were analyzed for relative DNA content by FACS on a FACScan flow cytometer based on red fluorescence.

### TUNEL assay

Apoptosis was measured by terminal deoxyribonucleotide transferase (TdT)-mediated dUTP nick-end labeling (TUNEL) detecting *in situ* DNA fragmentation. TUNEL staining was performed using an *in situ* Cell Death Detection Kit (Roche). In addition, a cell death detection ELISA plus kit (Boerhringer Mannheim, Indianapolis, IN, USA) was used to assess apoptotic activity by detecting fragmented DNA within the nuclei of drug-treated cells.

### Statistical analysis

Three or more separate experiments were performed, after which groups were compared by one way ANOVA. A p value of <0.05 was considered to indicate significance.

## Additional Information

**How to cite this article**: Jang, T.-H. *et al*. Rescuing neuronal cell death by RAIDD- and PIDD- derived peptides and its implications for therapeutic intervention in neurodegenerative diseases. *Sci. Rep.* **6**, 31198; doi: 10.1038/srep31198 (2016).

## Supplementary Material

Supplementary Information
