# Supplementary material for: Rescuing neuronal cell death by RAIDD- and PIDD- derived peptides and its implications for therapeutic intervention in neurodegenerative diseases
Source: Sci Rep. 2016 Aug 9;6:31198. doi: 10.1038/srep31198 (PMC4977500; doi:10.1038/srep31198)
Supplement: Supplementary Information [file srep31198-s1.pdf]

MICROBIOLOGY, BIOCHEMISTRY, STRUCTURAL BIOLOGY

*Hyun Ho Park, Phone: +82-53-810-3045. Fax: +82-53-810-4516. E-mail: hyunho@ynu.ac.kr
*Tae-Jin Lee, Phone: +82-53-640-6914. Fax:+82-53-621-5053. E-mail: tjlee@med.yu.ac.kr

Rescuing neuronal cell death by RAIDD- and PIDD- derived peptides and its implications for therapeutic intervention in neurodegenerative diseases

Tae-Ho Jang[a,#], In-Hye Lim[b,#], Chang Min Kim [a,#], Jae Young Choi[a], Eun-Ae Kim[b], Tae-Jin Lee[b,*] and Hyun Ho Park[a,*]

[a]School of Biotechnology and Graduate School of Biochemistry at Yeungnam University, Gyeongsan 38541, South Korea

[b]Department of Anatomy, College of Medicine, Yeungnam University, 317-1 Daemyung-Dong Nam-Gu, Daegu 42415, South Korea

Supplementary Figure S1

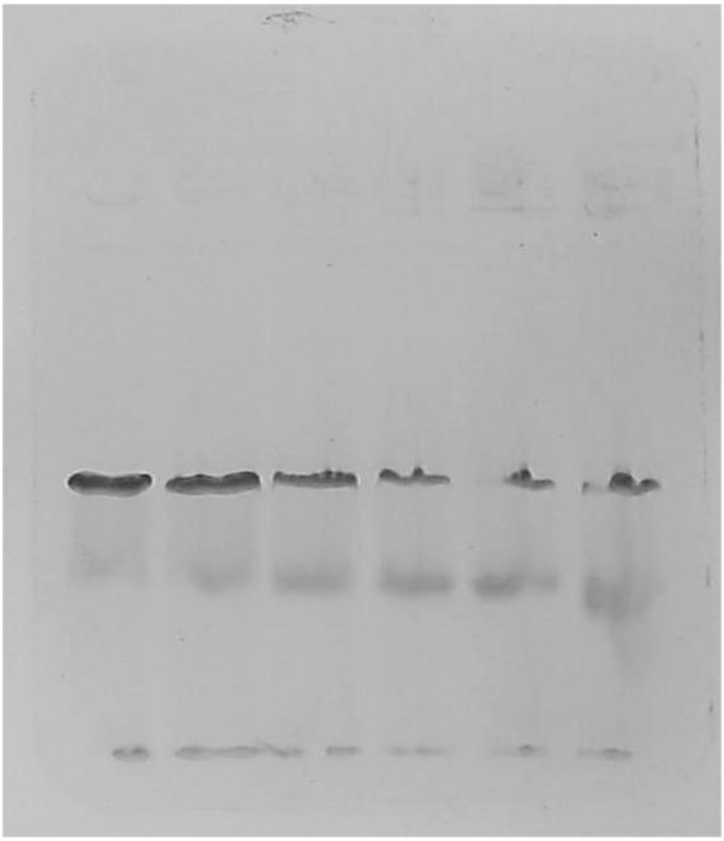

Figure S1. Peptide (PDH3) dose-dependent inhibition of PIDDosome core formation at low concentration range. The RAIDD DD:PIDD DD complex was prepared and mixed with the concentrations of PDH3 peptide indicated above the gel, after which the mixtures were subjected to native-PAGE.

Supplementary Figure S2

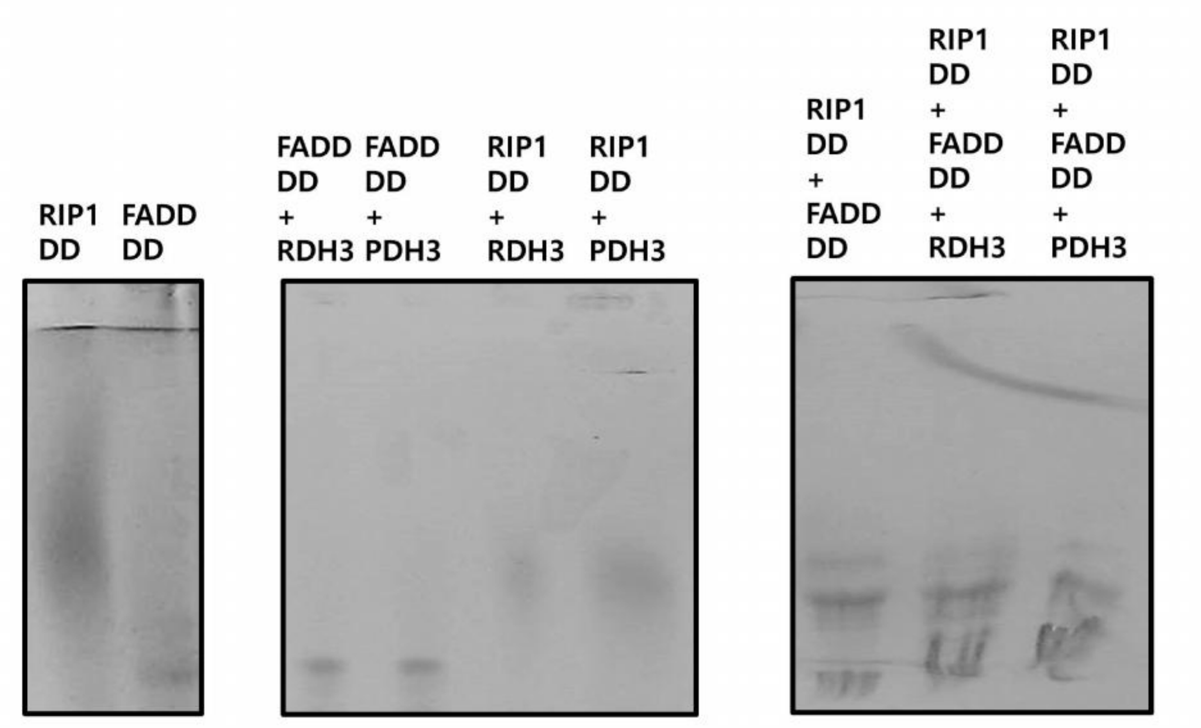

Figure S2. Peptides specificity analysis. Another DD family, RIP1 DD, FADD DD, was prepared for the peptides specificity analysis with Native-PAGE.
